# Supplementary material for: The interactome of intact mitochondria by cross-linking mass spectrometry provides evidence for coexisting respiratory supercomplexes
Source: Mol Cell Proteomics. 2017 Dec 8;17(2):216–32. doi: 10.1074/mcp.RA117.000470 (PMC5795388; doi:10.1074/mcp.RA117.000470)
Supplement: Supplemental Data [file supp_RA117.000470_133922_0_supp_23978_fzffwf.zip › spectra_annotation/mito_spectra_annotation/461-1-5-1-12-1.pdf]

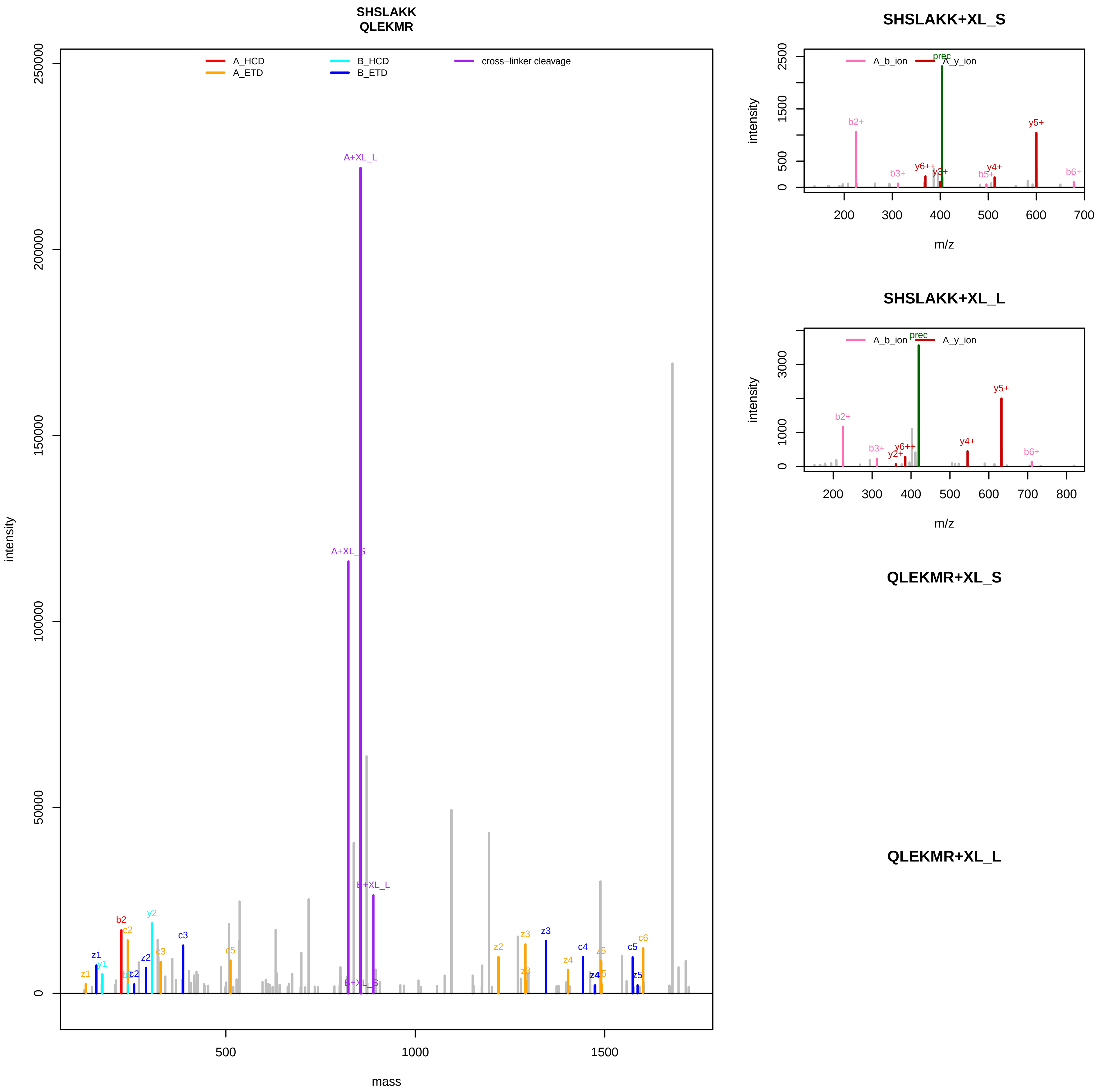
SHSLAKK
QLEKMR
A_HCD
A_ETD
B_HCD
B_ETD
cross−linker cleavage
A+XL_L
A+XL_S
B+XL_L
B+XL_S
intensity
mass
0
50000
100000
150000
200000
250000
500
1000
1500
z1
y1
b2
c2
b1
c2
z2
y2
c3
z3
c5
z2
z3
z3
c4
z4
z4
z5
c5
z5
c6
SHSLAKK+XL_S
A_b_ion
A_y_ion
prec
b2+
b3+
y6++
y3+
b5+
y4+
y5+
b6+
intensity
m/z
0
500
1500
2500
200
300
400
500
600
700
SHSLAKK+XL_L
A_b_ion
A_y_ion
prec
b2+
b3+
y2+
y6++
y4+
y5+
b6+
intensity
m/z
0
1000
3000
200
300
400
500
600
700
800
QLEKMR+XL_S
QLEKMR+XL_L
